# Blood alcohol concentration and self-reported alcohol ingestion in acute poisoned patients who visited an emergency department

**DOI:** 10.1186/1757-7241-21-24

**Published:** 2013-04-10

**Authors:** Seon Hee Woo, Woon Jeong Lee, Won Jung Jeong, Yeon Young Kyong, Se Min Choi

**Affiliations:** 1 Department of Emergency Medicine, College of Medicine, The Catholic University of Korea, Seoul, Republic of Korea; 2 Department of Emergency Medicine, Uijeongbu St. Mary's Hospital, Geumo-dong, Uijeongbu-si, Gyeonggi-do, Republic of Korea

**Keywords:** Poisoning, Alcohol drinking, Emergencies, Self-Report

## Abstract

**Background:**

Many acute poisoned patients have co-ingested alcohol in the emergency department (ED). This study aimed to estimate the blood alcohol concentration (BAC) of acute poisoned patients who visited an ED by age and gender distribution and to determine whether it is possible to obtain self-reports of alcohol ingestion among poisoned patients.

**Method:**

A retrospective medical chart review was conducted for all patients who visited the ED with acute poisoning between January 2004 and February 2008. Data regarding the patient’s age, gender, BAC, self-reported alcohol ingestion, poison ingested, time elapsed since poison exposure, presence of suicide attempts, and self-reported alcohol ingestion were collected. Patients were classified into two groups based on serum alcohol levels (≤10 mg/dl, >10 mg/dl).

**Results:**

Of the 255 subjects, 88 subjects (34.5%) were included in the non-alcohol group and 167 subjects (65.5%) were included in the alcohol group. 227 subjects (89.0%) showed suicide intention. Using the 201 subjects who completed the self-report of alcohol ingestion, self-report resulted in 96.6% sensitivity and 86.7% specificity for the assessment of alcohol ingestion. The positive and negative predictive values for self-report were 91.2% and 94.7%, respectively. The median (interquartile range) BAC of the 97 males in the sample was 85.0 (10.0-173.5) mg/dl, and that of the 158 females was 32.0 (4.0-137.5) mg/dl (p = 0.010). The distribution of age in the groups was significantly different between the alcohol and non-alcohol groups (p = 0.035), and there was a significant difference in the mean BAC with respect to age for males (p = 0.003).

**Conclusion:**

This study showed that over two-thirds of patients presenting with acute poisoning had a BAC > 10 mg/dl. Most of patients visited by suicide attempt. Males had a higher BAC than did females. Self-reported alcohol ingestion in acute poisoned patients showed high sensitivity and specificity.

## Background

Many acute poisoned patients have co-ingested alcohol in the emergency department (ED). Alcohol is quite accessible to the majority of the Korean population, and the drinking culture in the Republic of Korea is very liberal [1-3]. However, excessive alcohol ingestion not only causes medical complications but has also been identified as a risk factor for suicide and injury [4-7]. In acute poisoned patients, there is a high prevalence of intentional suicide attempts [8]. In addition, alcohol use at the time of a suicide attempt is quite common [7]. Among patients with alcohol dependence, the presence of a heavy drinking episode and being male were associated with a serious suicide attempt [9]. Finally, the early initiation of alcohol use is a risk factor for violent behaviors and suicide attempts among young adults [10]. While there are many reports that have evaluated alcohol and drug abuse, few studies have examined the clinical features of alcohol ingestion among acutely poisoned patients visiting the ED. Thus, it is important to evaluate alcohol ingestion among patients with acute poisoning.

Acute poisoned patients who presented to the ED may experience interactions between alcohol and poisonous substances. For example, antihistamines, antipsychotic drugs, tricyclic antidepressants, benzodiazepines, and opioids cause prolonged sedation, which can negatively impact the consciousness of acutely poisoned patients [11]. Therefore, the blood alcohol concentration (BAC) test is valuable in assessing alcohol ingestion when evaluating the consciousness of acutely poisoned patients. Unfortunately, it is difficult to use the BAC test in all poisoned patients. Other tests that are used to identify drunken drivers include the breath-alcohol test and self-reports of alcohol ingestion [12,13]. The BAC test and breath-alcohol test are difficult to use on poisoned patients due to the need for additional blood sampling and the patient’s lack of cooperation due to age or decreased mentation after poisoning. In contrast, the self-report of alcohol ingestion is rapidly obtained by taking a simple history. Also, previous report about injury patient founded that it has been shown to have high sensitivity and high specificity for detecting alcohol ingestion [13]. Therefore, we aimed to estimate the BAC of acutely poisoned patients who visited an ED by age and gender distribution and to evaluate the accuracy of self-reported alcohol ingestion.

## Methods

A retrospective study of acutely poisoned patients was conducted over a period of 50 months between January 2004 and February 2008 at the emergency department of Incheon Saint Mary’s Hospital in South Korea. Acutely poisoned patients who were older than 18 years of age and who had visited the ED within 10 hours of poisoning were included in the study. A total of 358 poisoned patients visited the ED during the period of this study. Of these, 45 subjects were excluded due to unclear or missing medical records. In addition, the study excluded 25 subjects who were transferred to our hospital after receiving gastric lavage at another hospital, 13 subjects who were transferred to another hospital from our hospital and 20 subjects who did not have a BAC level checked. Therefore, 255 subjects were included in this study. However, of the 255 patients, 54 lacked data (21.2%) on self-reported alcohol ingestion and were therefore excluded from this particular analysis. These included 5 subjects in the alcohol group and 49 subjects in the non-alcohol group. Of the excluded 54 subjects without self-reported alcohol ingestion, 30 had decreased mentation, with 25 were drowsy, 4 were stuporous and 1 was in a coma. This study was approved by the institutional review board of the Catholic University of Korea, Incheon Saint Mary’s Hospital.

After reviewing the patient’s medical charts, the patient’s age, gender, BAC, self-reported alcohol consumption, poisonous substances, elapsed time after exposure, and presence of a suicide attempt at the time of poisoning were collected. The BAC of subjects was measured from arterial or venous blood within an hour of arriving at our hospital. The BAC was measured using the DRI® Ethyl Alcohol Assay. The DRI® Ethyl Alcohol Assay can accurately quantitate BAC within a range from 10 mg/dl (10 mg/100 ml or 0.01 g%) to 600 mg/dl. Thus, subjects with an initial BAC higher than 10 mg/dl were included in the alcohol group, and those with a BAC lower than 10 mg/dl were included in the non-alcohol group.

A history of self-reported alcohol ingestion and elapsed time after exposure was collected from the patient or their guardian (e.g., parent, friend, sister, brother, witness) during the ED visit. The accuracy of self-reported alcohol consumption was assessed for its sensitivity, specificity, positive predictive value (PPV), and negative predictive value (NPV).

Statistical analyses were performed using SAS version 9.1 (SAS Institute, Inc, Cary, NC). The differences between the two groups were compared using Student’s *t* test, and the Mann–Whitney U test was used for continuous variables and expressed as median (interquartile range; IQR). The chi-squared and Fisher’s exact tests were used to assess categorical variables. Age, BAC and gender were analyzed using the Kruskal Wallis test. P values less than 0.05 were considered to be statistically significant.

## Results

### General characteristics of the poisoning patients

Of the 255 subjects included in the study, 97 were male (38.0%), and 158 were female (62.0%). Doxylamine was the most common substance of acute drug poisoning and was consumed by a total of 47 patients (Table 1). There were 88 subjects (34.5%) in the non-alcohol group and 167 subjects (65.5%) in the alcohol group. The prevalence of alcohol ingestion was significantly greater for males (p = 0.043). The median (IQR) ages of the subjects were 36 (27–51) and 41 (31–50) years of age for the non-alcohol group and the alcohol group, respectively (p = 0.119). The median (IQR) time between poisoning and the hospital visit was 2.0 (0.7-3.2) hours for the non-alcohol group and 1.3 (0.5-2.5) hours for the alcohol group. For 227 (89.0%) subjects, the poisoning was part of a suicide attempt, and the distribution of patients attempting suicide did not differ between the non-alcohol group and the alcohol group (p = 0.160) (Table 2).

**Table 1:** The materials of exposure for the acute poisoned patients

	**Non-alcohol group**	**Alcohol group**
	**(n, %)**	**(n, %)**
Doxylamine	18 (20.5)	29 (17.3)
Pesticides	12 (13.6)	22 (13.2)
Hypnosedatives	11 (12.5)	20 (12.0)
Antidepressants and antipsychotics	11 (12.5)	13 (7.8)
Acetaminophen and salicylates	8 (9.1)	11 (6.6)
Household products	6 (6.8)	26 (15.6)
Unknown, multi-mixed drugs or others	22 (25.0)	46 (27.5)
Total	88 (100)	167 (100)

**Table 2:** A comparison of the general characteristics of acute drug poisoned patients between alcohol and non-alcohol group

	**Non-alcohol group**	**Alcohol group**	**p-value**
	**(n = 88)**	**(n = 167)**	
Sex (n, %)			
Male	26 (26.8)	71 (73.2)	0.043
Female	62 (39.2)	96 (60.8)	
Age (years) ^*^	36 (27–51)	41 (31–50)	0.119
15-24 (n, %)	19 (21.6)	20 (12.0)	0.035
25-34 (n, %)	22 (25.0)	30 (18.0)	
35-44 (n, %)	19 (21.6)	57 (34.1)	
45-54 (n. %)	12 (13.6)	30 (18.0)	
55-64 (n, %)	4 (4.6)	16 (9.6)	
≥65 (n, %)	12 (13.6)	14 (8.4)	
Elapsed time after exposure (hour) ^*^	2.0 (0.7 – 3.2)	1.3 (0.5 – 2.5)	0.219
Suicide attempt (n, %)	75 (85.2)	152 (91.0)	0.160

^*^ Median value with interquartile range, statistical analyses were performed by Mann–Whitney U test.

**Figure 1:**
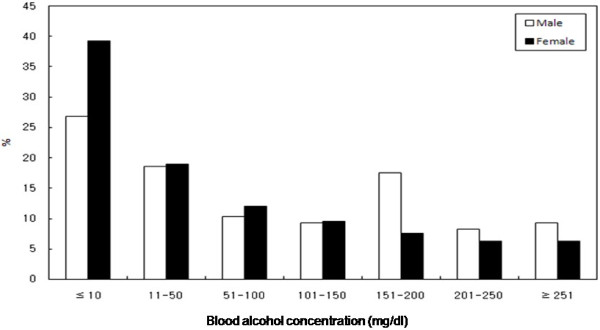
**The distribution of BAC in the acute poisoned patients.** 17 (17.5%) males and 20 (12.6%) females had a BAC exceeding 200 mg/dl. Over two-thirds of patients presenting had a BAC > 10 mg/dl. BAC: blood alcohol concentration.

After evaluating the distribution of the BACs of acutely poisoned subjects who visited the ED, 17 (17.5%) males and 20 (12.6%) females had a BAC exceeding 200 mg/dl. They had decreased mentation, with 32 were drowsy, 4 were stuporous and 1 was in a coma. Of the subjects, 26 (26.8%) males and 27 (17.1%) females had a BAC between 100 mg/dl and 200 mg/dl, 28 (28.9%) males and 49 (31.0%) females had a BAC between 10 mg/dl and 100 mg/dl (Figure 1). No subjects had a BAC exceeding 500 mg/dl. A total of 6 subjects died, and 3 of these subjects were in the alcohol group.

### Analysis according to self-reported alcohol ingestion

Of the 201 subjects with self-reported data, 118 (58.7%) had a BAC higher than 10 mg/dl. Of these 118 patients with a positive BAC, 114 confirmed that they had been drinking alcohol. Of the 76 subjects who denied alcohol ingestion, 4 patients had a positive BAC. Their mentation on presentation to the ED was as follows: 48 were alert, 150 were drowsy, and 3 were stuporous. Self-reported data were 96.6% sensitive and 86.7% specific for assessing alcohol ingestion. In addition, the PPV was 91.2%, and the NPV was 94.7%. The sensitivity was 98.2% in males and 95.2% in females, while specificity was lower in males than females (Table 3). The PPV was lower (88.7%) in males than in females (93.7%). The NPV was 94.7% for males and females.

**Table 3:** Gender and self-reported alcohol ingestion

**Self-report**	**Male (n = 81)**		**Female (n = 120)**	
	**BAC (+)**	**BAC (−)**	**BAC(+)**	**BAC (−)**
Drink alcohol (n)	55	7	59	4
Did not drink alcohol (n)	1	18	3	54
Sensitivity (%)	98.2		95.2	
Specificity (%)	72.0		93.1	
PPV (%)	88.7		93.7	
NPV (%)	94.7		94.7	

BAC: blood alcohol concentration, PPV: positive predictive value, NPV: negative predictive value.

### Distribution of BAC by gender and age

**Figure 2:**
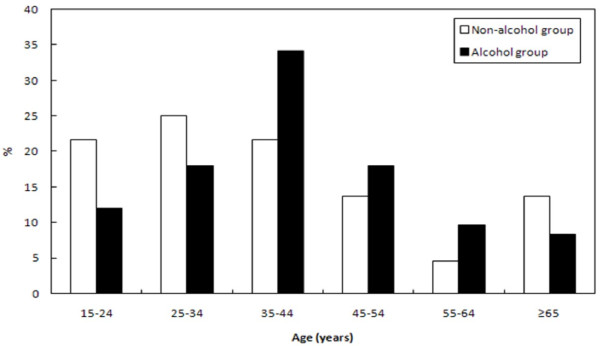
**The distribution of acute poisoned patients with respect to age.** We found that the distribution of age differed between the alcohol group and non-alcohol group (p = 0.035).

**Figure 3:**
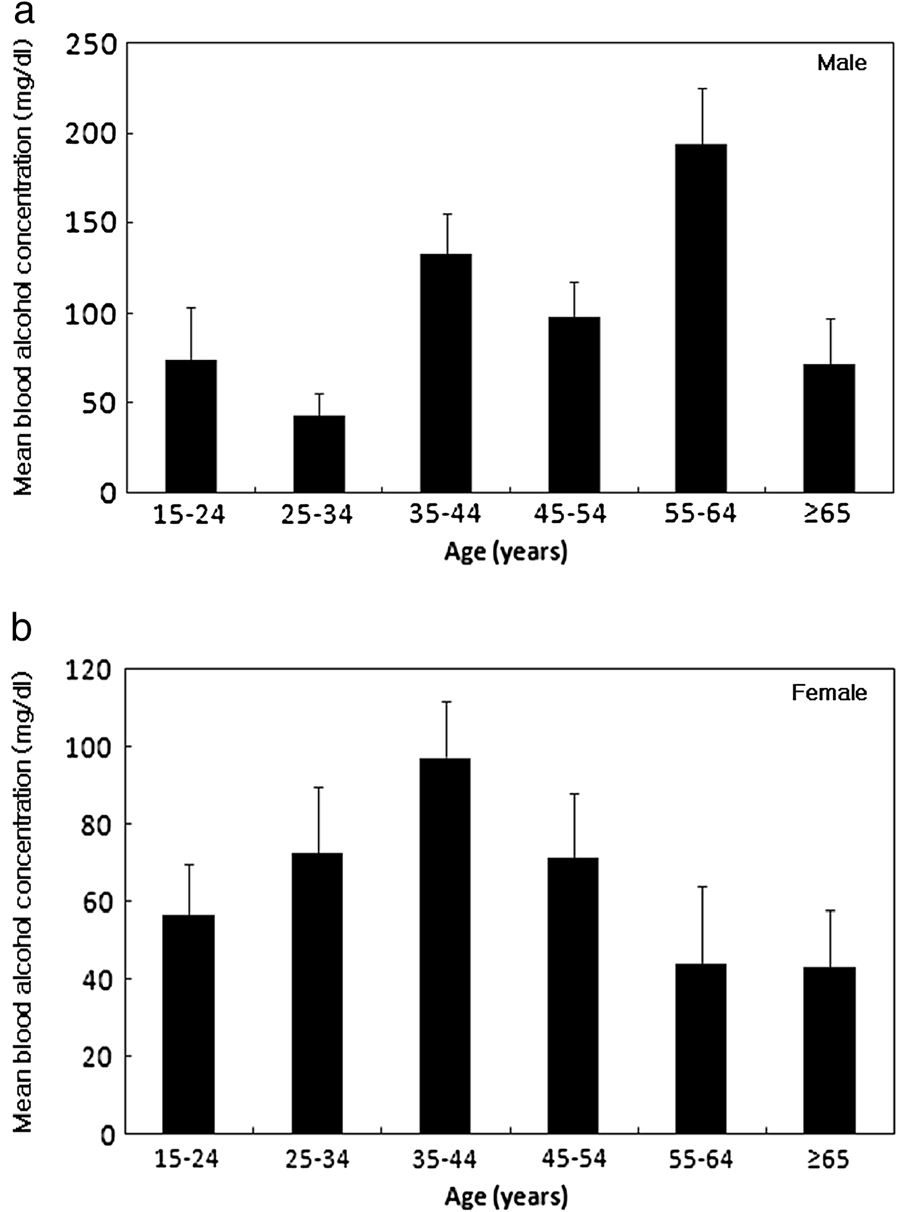
**The relationship between the mean BAC (BAC ± SE) and age for gender. (a)** For males, there was significant difference in the mean BAC with respect age (p = 0.003). A higher the mean BAC of males were showed in the age range 55–64 years age compared with age range 25–34 years age. **(b)** For females, there was no significant difference in the mean BAC with respect to age (p = 0.536). BAC: blood alcohol concentration; SE: standard error.

The mean BAC differed significantly between males and females. The median (IQR) BAC of the 97 males was 85.0 (10.0-173.5) mg/dl and that of the 158 females was 32.0 (4.0-137.5) mg/dl (p = 0.010). However, the median (IQR) BAC for males was 136.0 (50.0-198.0) mg/dl and that for females was 94.5 (47.0-172.3) mg/dl in the alcohol group (p = 0.223). We found that the distribution of age differed between the alcohol group and non-alcohol group (p = 0.035) (Figure 2). For males, there was significant difference in the mean BAC with respect age (p = 0.003). A higher the mean BAC of males were showed in the age range 55–64 years age compared with age range 25–34 years age. There was no significant difference in the mean BAC with respect to age for females (p = 0.536) (Figure 3).

## Discussion

Acute alcohol intoxication results in decreases in physical adaptability and judgment, thus increasing the risk of traffic accidents, falling, suicide, drowning, and fire [4,6,7,9]. Emergency room physicians see the majority of acutely poisoned patients who present to the ED following a suicide attempt or alcohol ingestion [8]. In our study, subjects with suicidal intentions accounted for 89.0% of the study population, although there were no differences between the non-alcohol group and the alcohol group. Alcohol may affect the potential lethality or severity of the suicide attempt [14]. However, we did not assess the relationship between the BAC and the severity of the suicide attempts among acutely poisoned patients. Our results did not suggest that alcohol drinking provoked unplanned suicidal poisonings by increasing anxiety and impulsivity. However, approximately two thirds of the poisoned patients had a BAC > 10 mg/dl, suggesting a high prevalence of alcohol ingestion among poisoned patients. Alcohol ingestion is popular in the Republic of Korea. In males, heavy drinking and binge drinking are common due to the predominant social culture surrounding alcohol in this country [1-3]. Females accounted for 62% of our study population; however, the rate of alcohol drinking and the median BAC were higher for males. This result may reflect the permissive attitude towards alcohol ingestion for males in the Republic of Korea.

The use of the BAC test may not helpful depending on the level of consciousness at arrival in ED, as there are many hospitals that are unable to offer the test because they are unable to get consent from the patient or their guardian. In addition, emergency physicians are often unable to promptly confirm the results of such testing due to delayed reporting and the need for evaluation by outside laboratories. In contrast, the use of the breath alcohol test and self-report of alcohol ingestion do not need laboratory analysis. Therefore, there is a need to evaluate the accuracy of self-reported alcohol ingestion in poisoned patients. The sensitivity and specificity of self-reported alcohol ingestion in trauma patients has been reported to be high. A report by Treno et al. showed that 87.1% of those testing positive for alcohol reported drinking prior to injury, and 93.1% of those testing negative reported no drinking [15]. Similarly, Sommers demonstrated that of 141 patients who had a BAC higher than 10 mg/dl, 134 self-reported drinking alcohol [13]. This result of 95% sensitivity similar 97% sensitivity of our study in self-reported alcohol ingestion. In our study, few acutely poisoned patients were in a coma. Thus, we suggests that obtaining self-reports of alcohol ingestion is possible in this sample. The lower specificity (86.7%) and PPV (91.2%) in our study is thought to be because patients were in the post absorption phase and the elimination rate of alcohol. After drinking on an empty stomach, the elimination rate of ethanol is 10 ~ 15 mg/dl per hour [16]. The patients that enrolled in our study visited the ED within 10 hours of poisoning; thus, in our study, BAC may have low specificity. Alcohol excretion and metabolism can vary based on the patient’s gender, age, body weight, stomach contents, and current use of medication [17].

Diagnosing alcohol ingestion using clinical data, e.g., abnormal gait, the smell of alcohol, slurred speech and eye redness has been reported to have low reliability [18]. Furthermore, it is more difficult to identify alcohol ingestion in a clinical examination of acutely poisoned patients in the ED, as such patients may also present with abnormal gait and slurred speech. The physician may be able to predict the level of alcohol ingestion by the smell alcohol; however, after the ingestion of pesticide or other toxic materials that can cause bad breath, it may be difficult to use this as an assessment of alcohol ingestion. According to our study, self-reported alcohol ingestion has a sensitivity of 96.9% and a specificity of 86.7%. Such high specificity and sensitivity demonstrated that the evaluation of self-reported alcohol ingestion is a reliable method of determining whether a patient has been drinking alcohol.

In trauma patients, self-reported alcohol ingestion differs among male and female traffic accident victims. Males may underreport the real amount and frequency of alcohol ingestion due to the resulting legal problems or impaired memory due to alcohol intoxication [13]. In our study, the self-report of males was slightly more sensitive than that of females. Patients were more likely to offer accurate self-reports because there was less risk of legal repercussions for acutely poisoned patients who present to the ED than for trauma patients. Therefore, a careful history of alcohol ingestion should be obtained from either the patient or their guardian while the patient is in the ED. This could help in the assessment of alcohol ingestion in acutely poisoned patients. However, self-reports of alcohol ingestion yield are limited in their ability to predict the BAC, which is important in assessing the level of consciousness. Moreover, in the uncooperative patient and those in a comatose state due to drugs or alcohol, self-reports of alcohol ingestion are not accurate or may be unfeasible. Thus, in acutely poisoned patients who are either uncooperative or comatose and for whom there is clinical suspicion that alcohol has been ingested, the BAC test must be considered.

The limitations of this study include its small sample size and the fact that patients were recruited from a single emergency department. In addition, given that BAC decreases over time or as the patient vomits, the BAC is subject to change within a pre-defined sample population (e.g., patients who arrive at the hospital within 10 hours of poisoning). Of the subject, 54 subjects had missing data, and their information was excluded from the analysis of the self-report of alcohol ingestion. Finally, we did not consider there are some laboratory differences between the arterial and venous samples in the BAC test [19]. Therefore, further prospective studies with large samples are needed.

## Conclusions

Over two-thirds of patients presenting with acute poisoning had a BAC > 10 mg/dl. Most of patients visited by suicide attempt. Males had a higher BAC than Females. Self-reported alcohol ingestion in patients with acute poisoning demonstrated 96.6% sensitivity and 86.7% specificity. In conclusion, our study suggests that self-reported alcohol ingestion could be helpful in the assessment of alcohol ingestion in acute poisoned patients.

## Abbreviations

BAC: Blood alcohol concentration; ED: Emergency department; IQR: Interquartile range; PPV: Positive predictive value; NPV: Negative predictive value.

## Competing interests

The authors decalre that they have no competing interests.

## Authors’ contributions

SHW performed data analysis and drafted the manuscript. WJL acquired data and critical revisions to the manuscript. WJJ and YYK managed the data and revisions to the manuscript. SMC conceived the research and drafted the manuscript. All authors have read and approved the final manuscript.
